# Functional Importance of Hydrophobic Patches on the Ebola Virus VP35 IFN-Inhibitory Domain

**DOI:** 10.3390/v13112316

**Published:** 2021-11-20

**Authors:** Nodoka Kasajima, Keita Matsuno, Hiroko Miyamoto, Masahiro Kajihara, Manabu Igarashi, Ayato Takada

**Affiliations:** 1 Division of Global Epidemiology, International Institute for Zoonosis Control, Hokkaido University, Sapporo 001-0020, Japan; kasajima@czc.hokudai.ac.jp (N.K.); hirom@czc.hokudai.ac.jp (H.M.); kajihara@czc.hokudai.ac.jp (M.K.); 2 Division of Risk Analysis and Management, International Institute for Zoonosis Control, Hokkaido University, Sapporo 001-0020, Japan; matsuk@czc.hokudai.ac.jp; 3 Global Station for Zoonosis Control, Global Institution for Collaborative Research and Education, Hokkaido University, Sapporo 001-0020, Japan; 4 One Health Research Center, Hokkaido University, Sapporo 001-0020, Japan; 5 School of Veterinary Medicine, The University of Zambia, Lusaka P.O. Box 32379, Zambia

**Keywords:** Ebola virus, VP35, polymerase cofactor, interferon antagonist, IFN-inhibitory domain, hydrophobic patch

## Abstract

Viral protein 35 (VP35) of Ebola virus (EBOV) is a multifunctional protein that mainly acts as a viral polymerase cofactor and an interferon antagonist. VP35 interacts with the viral nucleoprotein (NP) and double-stranded RNA for viral RNA transcription/replication and inhibition of type I interferon (IFN) production, respectively. The C-terminal portion of VP35, which is termed the IFN-inhibitory domain (IID), is important for both functions. To further identify critical regions in this domain, we analyzed the physical properties of the surface of VP35 IID, focusing on hydrophobic patches, which are expected to be functional sites that are involved in interactions with other molecules. Based on the known structural information of VP35 IID, three hydrophobic patches were identified on its surface and their biological importance was investigated using minigenome and IFN-β promoter-reporter assays. Site-directed mutagenesis revealed that some of the amino acid substitutions that were predicted to disrupt the hydrophobicity of the patches significantly decreased the efficiency of viral genome replication/transcription due to reduced interaction with NP, suggesting that the hydrophobic patches might be critical for the formation of a replication complex through the interaction with NP. It was also found that the hydrophobic patches were involved in the IFN-inhibitory function of VP35. These results highlight the importance of hydrophobic patches on the surface of EBOV VP35 IID and also indicate that patch analysis is useful for the identification of amino acid residues that directly contribute to protein functions.

## 1. Introduction

Ebola virus (EBOV) is an enveloped, negative-stranded RNA virus that belongs to the genus *Ebolavirus* in the family *Filoviridae*. The genus *Ebolavirus* consists of six species represented by EBOV, Sudan virus (SUDV), Taï Forest virus (TAFV), Bundibugyo virus (BDBV), Reston virus (RESTV), and Bombali virus (BOMV) [1,2]. The genus *Marburgvirus*, other principal members of the virus family, includes two viruses, Marburg virus (MARV) and Ravn virus (RAVV) in a single species. EBOV, SUDV, TAFV, BDBV, MARV, and RAVV cause hemorrhagic fever in humans and nonhuman primates with high mortality rates of up to 90%, for which clinically approved antivirals and vaccines remain limited [3]. In addition, other filoviruses such as Lloviu virus (LLOV) and Měnglà virus (MLAV) have been recently discovered and classified into the genera *Cuevavirus* and genus *Dianlovirus*, respectively, in the family *Filoviridae* [4,5].

EBOV has a non-segmented RNA genome (approximately 19 kb) encoding three nonstructural and seven structural proteins [6]. Among the viral structural proteins, viral protein 35 (VP35), viral protein 30 (VP30), nucleoprotein (NP), and RNA-dependent RNA polymerase L protein act as essential components of the viral replication complex. EBOV VP35 is also known as an interferon (IFN) antagonist that interacts with host proteins involved in multiple IFN-production pathways and multiple IFN-stimulated genes (ISGs) [7]. VP35-mediated suppression of IFN production involves a variety of host factors: VP35 inhibits IFN production by interacting with TANK-binding kinase 1 (TBK1) and inhibitor of kappa B kinase epsilon (IKKε) and by suppressing post-translational modifications of IFN regulatory factors (IRF)-3 and -7 [8].

VP35 is composed of two major domains, the N-terminal coiled-coil domain and C-terminal IFN inhibitory domain (IID) [9]. On one hand, the coiled-coil domain is essential for several VP35 functions, including viral genome replication and nucleocapsid formation [10]. On the other hand, IID is required and sufficient for binding to dsRNA, leading to IFN inhibition [11,12,13]. IID has a cluster of conserved basic amino acid residues that are important for dsRNA binding [14]. IID is also sufficient to interact with NP [15]. Previous studies on the VP35 IID structure have defined two highly conserved basic regions, designated “first basic patch” and “central basic patch,” both of which are important for the VP35 functions [14,15]. The first basic patch consisting of residues K222, R225, K248, and K251 is critical for both polymerase cofactor function and the interaction with NP [15,16] while the central basic patch consisting of R305, K309, R312, R319, R322, and K339 is critical for IFN-antagonism [14,16]. Amino acid substitutions in these patches resulted in increased IFN-α/β responses, reduced viral replication, and attenuation of EBOV in animal models [14,17]. Thus, these basic patches in IID are thought to be important for both polymerase cofactor activity and IFN antagonism [15,18].

Protein patches generally reflect physical properties of molecular surfaces of protein structures and patch analyses are used to predict surface regions involved in protein-protein interactions [19]. Among protein patches on protein surfaces defined by physical properties (solvation potential, residue interface propensity, hydrophobicity, planarity, protrusion, and accessible surface area), hydrophobic patches are particularly expected to be functional sites that are involved in interactions with other proteins [19,20]. In the present study, we identified three hydrophobic patches on the IID surface using the 3D structural information of the VP35 molecule (PDB ID: 3FKE). One of the hydrophobic patches was found to be critical for the VP35 function as a viral polymerase cofactor. We further found that a subset of amino acid residues located within this hydrophobic patch was important for the VP35-NP interaction, which is required for formation of the genome replication complex. Furthermore, two of the three hydrophobic patches were also found to be important for the suppression of IFN production by VP35. Our data highlight the importance of the IID hydrophobic patches in the principal functions of EBOV VP35.

## 2. Materials and Methods

### 2.1. Patch Analysis

Protein surface patches of EBOV (Variant Mayinga, species *Zaire ebolavirus*) VP35 IID (PDB code: 3FKE, chain B) were detected using Molecular Operating Environment (MOE) software (version 2018; Chemical Computing Group, Montreal, QC, Canada). Three hydrophobic patches were identified on the VP35 IID structure. To experimentally investigate whether these patches were functionally important, amino acid substitutions that eliminated each hydrophobic patch were determined by computational calculations with MOE. When considering a patch consisting of *n* residues, *n* × 19 mutants were generated at each patch by mutating a residue to the other 19 amino acids in silico. Then, for these mutants, we calculated the difference in the patch area and thermostability (dStability) from wild-type VP35 to each mutant (Tables S1–S3). Finally, we chose the mutants having the following three characteristics: (i) disappearance of the relevant patch area, (ii) no effects on other patches (i.e., numbers and area), and (iii) dStability within 2.0 kcal/mol.

### 2.2. Cell Culture and Construction of Plasmids

Human embryonic kidney (HEK) 293 (ATCC1 CRL-1573™) and HEK293T cells (ATCC1 CRL-3216™) were grown in Dulbecco’s modified Eagle’s medium with 10% fetal calf serum (FCS). The cells were incubated in a humidified 5% CO2 incubator at 37 °C. The cDNA encoding HA-tagged VP35 of an EBOV variant, Mayinga (species *Zaire ebolavirus*), were cloned into the mammalian expression vector pCAGGS [21] as described previously [22]. HA-tagged mutant VP35 genes were also constructed by site-directed mutagenesis using KOD One (Toyobo) and cloned into pCAGGS. The NP, VP35, VP30, VP24, and L genes of EBOV (Mayinga) were similarly cloned into pCAGGS. An EBOV minigenome plasmid containing the firefly luciferase gene, p3E5E-luc, was constructed as described previously [22,23].

### 2.3. Minigenome Reporter Assay

The EBOV minigenome assay was carried out using a previously described system [22,24]. Briefly, HEK293T cells (2 × 10^5^) on 24-well plates were transfected with 50, 100, or 200 ng of plasmids encoding HA-tagged wild-type EBOV VP35, mutant VP35, or the HA tag alone, along with the plasmids for the expression of EBOV NP (50 ng), VP30 (30 ng), L (400 ng), p3E5E-luc (100 ng), and the T7 polymerase (100 ng) using the polyethylenimine (PEI) reagent. At 36 h after transfection, the cells were lysed with Passive Lysis Buffer (Promega), and the luciferase activity was measured using the Dual-Glo luciferase assay system (Promega) according to the manufacturer’s instructions. These cell lysates were also subjected to sodium dodecyl sulfate-polyacrylamide gel electrophoresis (SDS-PAGE), followed by Western blot analysis to examine the expression of β-actin and VP35 with an anti-β-actin (Abcam, ab6276) and anti-HA monoclonal antibodies (Abcam, ab1424) diluted at 1:5000. The firefly luciferase activity was compared to a negative control (i.e., absence of HA tagged-VP35) to obtain fold luciferase activity values.

### 2.4. Immunoprecipitation Assay

HEK293T cells were transfected with the pCAGGS plasmids encoding HA-tagged VP35 and NP using the PEI reagent according to the manufacturer’s instructions. At 36 h after transfection, the cell lysates were prepared with cold lysate buffer (50 mM Tris-HCl, pH 8.0, 150 mM NaCl, 2 mM EDTA, 10% glycerol, and 0.05% NP-40) containing EDTA-free protease inhibitors (Roche). The cell lysates were subsequently mixed with EZview Red Anti-HA Affinity Gel beads (Sigma) and incubated at 4 °C overnight with gentle rocking. After washing with lysate buffer, precipitated proteins were subjected to SDS-PAGE and Western blot analyses with a monoclonal anti-HA antibody (Abcam, ab1424) diluted at 1:5000 and rabbit antiserum to EBOV NP (FS0169) [25] diluted at 1:2000. The bound antibodies were visualized using Immobilon Western (Millipore). Band intensities were quantified using Amersham Imager 600 Analysis Software.

### 2.5. IFN-β Promoter Reporter Assay

HEK293 cells (2 × 10^5^) on 24-well plates were transfected with the HA-tagged VP35-expressing plasmid along with the plasmids for the human IFN-β promoter-driven firefly luciferase reporter gene (pIFNβ-luc., kindly gifted by Sonja Best, NIH/NIAID) and for the Renilla luciferase cloned into pRL-TK vector (Promega). Twenty-four hours after transfection, the cells were stimulated with 5 ng/µL poly(I:C) (InvivoGen). The cells were lysed in Passive Lysis Buffer (Promega), and then luciferase assays were performed using the Dual-Luciferase Reporter Assay System (Promega) according to the manufacturer’s directions. Firefly luciferase values were normalized to Renilla luciferase values. Normalized values were then compared to a negative control (no induction by poly(I:C)) to obtain fold induction values. Samples were also used for Western blotting as described above.

### 2.6. Statistical Analysis

Statistical analyses were carried out using Dunnett’s multiple-comparison test and the Tukey-Kramer test implemented in R [26]. Significance was defined as a *p*-value of less than 0.05 (* *p* < 0.05, ** *p* < 0.01, *** *p* < 0.001 or † *p* < 0.05, †† *p* < 0.01, ††† *p* < 0.001).

## 3. Results

### 3.1. Hydrophobic Patches Present on the Surface of VP35 IID and Amino Acid Substitutions to Modify the Patch Properties

**Figure 1:**
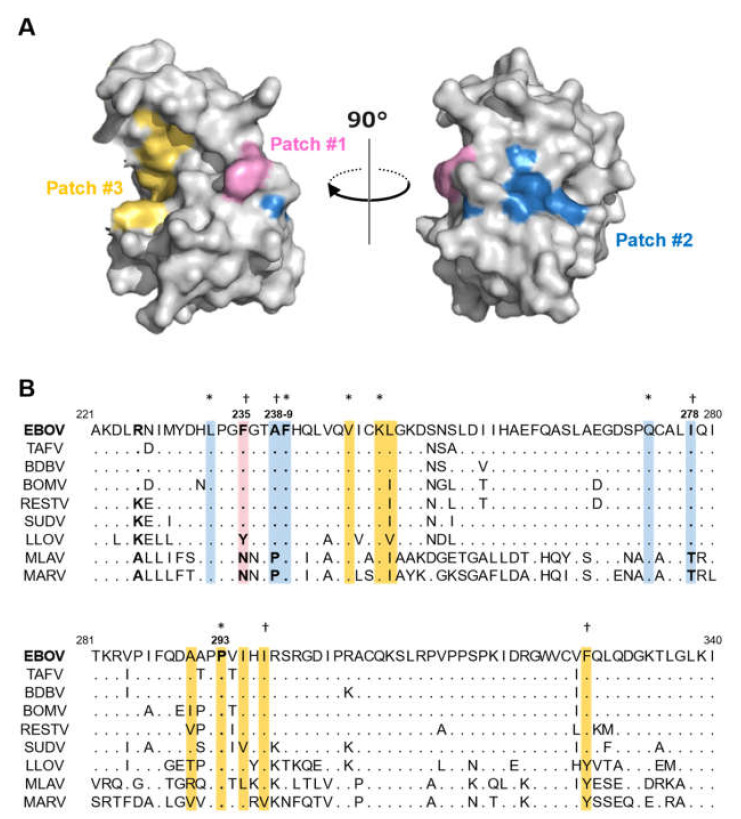
Hydrophobic patches on the VP35 IID surface (**A**) Three hydrophobic patches are shown on a structural model of VP35 IID (PDB ID: 3FKE). Patch #1 contains a single residue F235 (red). Patch #2 contains residues L232, A238, F239, Q274, and I278 (blue). Patch #3 contains residues V245, K248, L249, A290, P293, I295, I297, and F328 (yellow). (**B**) Multiple amino acid sequence alignment of VP35 IID among filovirus species. Highlighted amino acids represent those involved in the respective hydrophobic patches (red: patch #1, blue: #2, yellow: #3). Amino acids that are conserved among filoviruses and ebolaviruses are indicated by asterisks and daggers, respectively.

To test the hypothesis that hydrophobic regions of IID are important for the polymerase cofactor function and IFN-antagonist activity of VP35, we first aimed to identify hydrophobic regions (i.e., hydrophobic patches) on the surface of EBOV VP35 IID. Using in silico analysis, we found three regions that had hydrophobic properties on the IID surface (Figure 1A). These hydrophobic patches consisted of multiple amino acid residues: Patch #1 consisting of F235, patch #2 consisting of L232, A238, F239, Q274, and I278, and patch #3 consisting of V245, K248, L249, A290, P293, I295, I297, and F328. Some of these amino acid residues were located in the hydrophobic region that has been previously described [14,16,27]. Particularly, F239 has been previously described as an essential amino acid that is important for dsRNA binding and suppression of the IFN-β promoter activation, and K248 belonging to the basic patch has been shown to be responsible for the VP35-NP interaction [15,16]. We then compared corresponding amino acid residues among filoviruses (Figure 1B). Most of the amino acids comprising the hydrophobic patches were shared among ebolaviruses but only partially with LLOV, MLAV, and MARV. Consistent with a previous study [16], it was noted that several amino acids such as F239 and P293 were conserved among all filoviruses (Figure 1B). We hypothesized that these hydrophobic patches might act as functional sites of VP35 and investigated the biological importance of each amino acid residue in the following experiments. For this purpose, we first sought amino acid substitutions that had little effect on the overall stability of the VP35 IID but eliminated the hydrophobic patches. For each amino acid that constituted the hydrophobic patches, the area size of the patch, the effect on other patches, and the stability of the patch were calculated when substituted for by 19 other amino acids in silico. We identified 15 substitutions at five positions (235, 238, 239, 278, and 293) (Table 1). Then plasmids for the expression of wild-type VP35, the previously known dysfunctional VP35 R225E mutant [15], and 15 VP35 mutants with the identified substitutions were constructed.

### 3.2. Reduced Function as a Polymerase Cofactor in Patch-Disrupted VP35 Mutants

**Figure 2:**
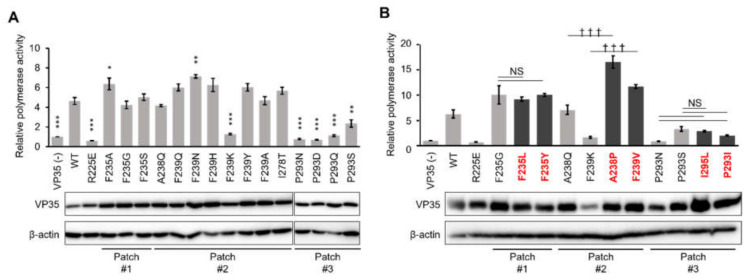
Attenuation of polymerase cofactor activity of hydrophobic patch-disrupted mutants of VP35. Viral polymerase activity was assessed by minigenome assay. HEK293T cells were transfected with an empty vector (VP35(-)), WT-, or mutant VP35-expressing plasmids together with expression plasmids for EBOV NP, VP30, L, T7 RNA polymerase, and a plasmid providing the EBOV minigenome encoding a fused firefly luciferase reporter gene under the control of the T7 RNA polymerase promoter. A Renilla luciferase-expression plasmid was cotransfected as a control for transfection efficiency. Minigenome activity was quantified by measuring firefly luciferase activity, and this was normalized to the level of Renilla luciferase expression. Each patch-disrupted mutant was analyzed to compare with wild-type VP35 (**A**) and patch-retained mutants (**B**). Each bar represents mean ± standard error (SE) for three independent experiments. Lower panels show Western blots for VP35 proteins and β-actin. Mutants shown in red boldface (also shown with dark bars) are those that retained the hydrophobic patch. Dunnett’s multiple-comparison test was used for the comparison to wild-type (WT) (* *p* < 0.05, ** *p* < 0.01, *** *p* < 0.001) (**A**). The Tukey–Kramer test was used for comparisons between the patch-disrupted and patch-retained mutants in each amino acid position (††† *p* < 0.001, NS: No significant difference) (**B**).

Since VP35 is required for EBOV genome replication as a viral polymerase cofactor [15,28,29], the modification of VP35 might affect polymerase activity. To investigate the functionality of VP35 for the EBOV genome transcription and replication, a plasmid-based EBOV minigenome assay was used [24]. HEK293T cells were cotransfected with the EBOV minigenome plasmid encoding the firefly luciferase and expression plasmids encoding the T7 RNA polymerase, EBOV L, NP, VP30, and VP35. The luciferase activity in the cell lysate was then analyzed (Figure 2A). The expression of the luciferase from the EBOV minigenome was significantly impaired by F239K substitution in patch #2 and all of the substitutions tested in patch #3 (positions 293 and 295), but no significant difference in the VP35 expression in the transfected cells was observed in immunoblotting. On the other hand, none of the substitutions in patch #1 significantly altered the luciferase expression compared to that in the presence of wild-type VP35. These results suggested that disruption of hydrophobic patch #2 and patch #3 decreased viral replication and that the hydrophobic regions of IID were important for the polymerase cofactor activity of VP35. The subcellular localization of the representative VP35 mutants in plasmid-transfected HEK293T cells was found to be similar to that of the wild-type in immunofluorescence assays (Figure S1).

To confirm the importance of the hydrophobic properties of patches #2 and #3 for the polymerase cofactor function, we tested VP35 mutants having amino acid substitutions that did not impair the hydrophobic patches and had little effect on the protein structure in the patch analysis (Table 1 and Figure 2B). As expected, none of the amino acid substitutions in patch #1 (F235L and F235Y) significantly reduced the luciferase activity compared to the F235G mutant. In contrast, the mutants that retained the properties of patch #2 (A238P and F239V) showed higher polymerase cofactor activity than the patch-disrupted mutants (A238Q and F239K, respectively). For two patch-retained mutants of patch #3 (I295L and P293I), there was no significant difference in polymerase cofactor activities compared to those of the patch-disrupted mutants (P293N and P293S), suggesting that disruption of the hydrophobic property of patch #3 was not the principal determinant for the reduction of the polymerase cofactor function of VP35. These results suggested that the hydrophobic property of patch #2 on VP35 IID was important for the polymerase cofactor function of VP35.

### 3.3. Reduced Interaction between NP and Patch-Disrupted VP35 Mutants Having Lower Polymerase Cofactor Activity

**Figure 3:**
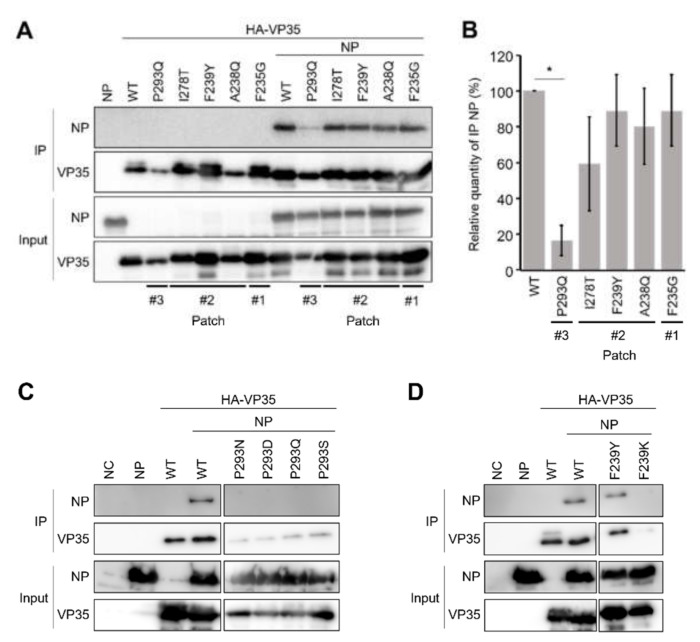
NP-VP35 interaction attenuated by hydrophobic patch modifications in VP35 IID. (**A**,**C**,**D**) Representative western blot images of EBOV NP immunoprecipitated from HEK293T cells transfected with the plasmids expressing EBOV NP and with wild-type VP35 (WT) or VP35 mutants. Representative mutants for patches #1, #2, and #3 (**A**), patch #3-disrupted mutants (**B**), and F239 mutants (patch #2) (**C**) were analyzed. Cells were lysed with a lysate buffer 36 h post-transfection. Then NP-bound HA-tagged VP35 was precipitated from samples using anti-HA affinity gel beads and analyzed by Western blotting. (**B**) Band intensities of immunoprecipitated NP relative to VP35 are quantified by western blotting. Each bar represents mean ± SE from three independent experiments. Dunnett’s multiple-comparison test was used to compare to WT (* *p* < 0.05).

The interaction between VP35 and NP is essential for EBOV polymerase activity. Previously, IID was shown to be sufficient for the interaction with NP. It was also shown that amino acid residues in one of the basic patches, consisting of residues K222, R225, K248, and K251, were critical for both polymerase cofactor function and the interaction with NP [15,16]. To determine whether the modification of the hydrophobic patches could affect the VP35-NP interaction, VP35 (wild-type and mutants) and NP were expressed in HEK293T cells and their interactions were analyzed by immunoprecipitation assays (Figure 3A,B). We first selected the most stable patch-disrupted mutants (i.e., those with the lowest *dStabilities* predicted by the patch analysis shown in Table 1) in each amino acid position (F235G for #1 and #2: A238Q, F239Y, and I278T for #2, and P293Q for #3) predicted by the patch analysis (Table 1) for this experiment. It was found that the interaction between VP35 and NP was significantly weakened by the P293Q substitution in patch #3 but not by the mutations in patch #1 and #2. To confirm the importance of patch #3 in the VP35-NP interaction, the other patch #3 mutants were also tested in immunoprecipitation assays (Figure 3C). We found that the amounts of NP immunoprecipitated with these patch #3 mutants were at almost undetectable levels although the band intensities of immunoprecipitated patch #3 VP35 mutants were weaker than that of wild-type VP35. We also tested the patch #2 F239K mutant, which showed low polymerase cofactor activity, and found that NP was not immunoprecipitated (Figure 3D). However, since the mutant was less effectively immunoprecipitated than wild-type VP35, it was not fully clarified whether the low polymerase cofactor activity was due to reduced interaction between NP and the F239K mutant. It is conceivable that the substitution might cause decreased solubility and stability of the protein, resulting in reduced immunoprecipitation efficiency.

### 3.4. Decreased IFN Antagonism of Patch-Disrupted VP35 Mutants

**Figure 4:**
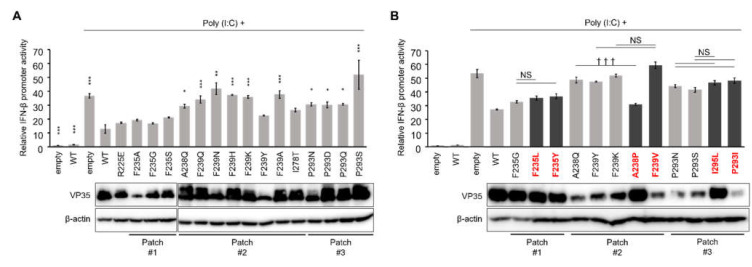
Antagonism of IFN production attenuated by hydrophobic patch modifications in VP35 IID. IFN-β promoter activity was assessed for patch-disrupted mutants alone (**A**) and together with patch-retained mutants (**B**) in the reporter assay. HEK293 cells were transfected with an empty vector (empty), WT-, or mutant VP35-expressing plasmids, and expression plasmids of Renilla luciferase along with pIFNβ-luc. IFN-β promoter was activated by poly(I:C) stimulation. IFN-β promoter activity was quantified by measuring luciferase and normalized to the level of expression of Renilla luciferase. Error bars represent 1 standard error (SE) for three independent experiments. Lower panels show western blots for VP35 proteins and β-actin. Mutants shown in red boldface (also shown with dark bars) are those that retain the hydrophobic patch. Each bar represents mean ± SE from three independent experiments. Dunnett’s multiple-comparison test was used for the comparison to WT stimulated with poly(I:C) (* *p* < 0.05, ** *p* < 0.01, *** *p* < 0.001) (**A**) and the Tukey–Kramer test for comparisons between the mutants (i.e., patch-disrupted and patch-retained mutants in each amino acid position (††† *p* < 0.001, NS: No significant difference) (**B**).

VP35 inhibits the RIG-I pathway for IFN production at multiple steps. For example, it acts as a suppressor of the cellular kinases IKKε and TBK1 [8,12]. The VP35 IID is thought to be required for suppression of IFN-α/β gene expression [17]. Thus, we next analyzed the effect of the patch modification on the inhibitory activity of VP35 with regard to IFN-β activation. HEK293 cells were cotransfected with the reporter plasmids carrying the IFN-β promoter and luciferase genes, and the expression plasmids for VP35 and Renilla luciferase. Then IFN production was induced by poly(I:C) stimulation and IFN-β promoter activity was determined by a reporter assay (Figure 4). As previously reported, wild-type VP35 inhibited IFN-β promoter activation. Consistent with the previous finding [16], we found that the patch #1 mutants showed the same level of suppression of the IFN-β promoter activity as wild-type VP35 even though the expression levels of the mutants were almost the same as or lower than that of wild-type VP35. On the other hand, the patch #2 and #3 mutants showed little effect on the reduction of IFN-β promoter activity compared to wild-type VP35, although the expression levels of the mutants were almost the same as or lower than that of wild-type VP35, except for the F239Y mutant. Interestingly, some patch #2 mutants (A238Q, F239Q, F239N, and F239A) that did not affect the polymerase cofactor activity reduced the ability to suppress the IFN-β promoter activity (Figure 4A). The same experiment was carried out with the mutants that were predicted to have less significant changes in their patch properties (Figure 4B). Unexpectedly, there were no significant differences between the patch-disrupted mutants and the patch-retained mutants, except for A238P. The IFN-β promoter activity was significantly reduced by one of the patch #2 mutants (A238P), which was comparable to wild-type VP35. Since the expression level of the A238P mutant was higher than those of the other patch #2 mutants, it could not be ruled out that the strong inhibition of IFN-β promoter activity by A238P might have been due to this difference. However, the expression levels of VP35 did not generally correlate with the suppression of IFN-β promoter activity. These findings suggested that amino acid residues in patches #2 and #3 of VP35 were important for the suppression of IFN production although the patch hydrophobicity was not the only factor for the functional importance.

## 4. Discussion

Previous studies have provided insights into the structure and biological activity of EBOV VP35, which functions as a virulence factor that suppresses innate immunity and as a polymerase cofactor that is essential for viral RNA replication/transcription [14,15,18,27]. Although it has been shown that the VP35 function as a part of the viral polymerase complex appears to require its interaction with viral NP and L [29,30] and that the C-terminal IID of VP35 is essential for both interaction with NP and antagonism of IFN production [15]. The present study focused on the hydrophobic patches present on the surface of VP35 IID and further characterized the properties of the domain using an in silico analysis followed by site-directed mutagenesis in biological assays (Table S4).

The hydrophobic patches identified in the present study are partly overlapped with the functional region described previously in the dsRNA end-capping hydrophobic pocket and the first basic patch, and some amino acid residues important for the VP35 functions have been identified in the previous studies [15,16]. Amino acid residues F235 and F239 on VP35 IID, which were predicted to form hydrophobic patches in our patch analysis, have been previously found to be functional amino acids by using alanine scanning mutagenesis [15,16]. The F235A substitution, but not F239A, was previously demonstrated to lose function as a polymerase cofactor [16]. However, in our experiments, while the F239A mutant retained the same level of polymerase cofactor function as wild-type VP35, the F235A mutant did not show significant loss of polymerase cofactor activity, inconsistent with previous reports. (Figure 2A). The discrepancy may be due to the difference in experimental conditions, sensitivity, and/or evaluation methods. However, considering that both F235A and F239A mutants were shown to retain the ability to bind to NP [15,16], we assume that the F235A mutant does not completely lose its polymerase cofactor activity. In this study, the effects of F235A and F239A substitutions on IFN antagonism were similar to those found in a previous study [16]; the F235A mutant had the ability to suppress IFN production as well as wild-type VP35, whereas the F239A mutant had reduced ability to suppress IFN production. These data suggest that our in silico patch analysis may be a useful tool to identify functional amino acids on molecular surfaces of proteins.

Although alanine scanning mutagenesis has been generally used to identify amino acid residues critical for protein functions, it was indeed unclear whether mutations to alanine fully altered the functions of the relevant proteins and whether each alanine mutation affected the overall stability of molecules. In this study, therefore, in silico analysis was performed to predict which amino acid residues could be used for substitutions that would disrupt each hydrophobic patch but not affect the protein structure. In patch #2, among the mutations at position 239, only the F239K mutation greatly reduced the polymerase cofactor activity. This finding suggests an important role of the hydrophobicity in this VP35 function whereas it might also be possible that other factors such as the difference in the properties of these amino acid residues caused a structural change that might disrupt the polymerase cofactor function. As mentioned above, both in a previous report and in the present study, the F239A mutant showed polymerase cofactor activity comparable to that of wild-type VP35. This suggests that mutations to alanine are not necessarily sufficient to fully affect protein functions, and that substitutions to other amino acids should be considered for mutagenesis studies. On the other hand, it should also be noted that some amino acid substitutions may alter protein oligomerization or stability status, which potentially affect expression levels of proteins. This might be one of the reasons for inconsistent VP35 band intensities seen in this study.

In this study, we have shown for the first time that P293 of VP35 contributes to polymerase cofactor activity and IFN-β suppression. Using computer analyses, the P293 residue has been predicted to be important for the interaction with small molecule compounds that may potentially inhibit VP35 functions [31,32,33]. However, the actual importance of this amino acid residue had not been evaluated in biological assays *in vitro*. In this study, we provide direct evidence that P293 is important for both polymerase cofactor function and suppression of IFN production, supporting the previous in silico studies that predicted the functional importance of this amino acid position [31,32,33]. Interestingly, the VP35 functions were impaired even by the P293 mutants with amino acid substitutions that had little effect on the patch #3 hydrophobicity (Figure 2). This suggests that the unique properties of the proline residue at this position, as well as the hydrophobic feature of patch #3, are also important for VP35 functions.

**Figure 5:**
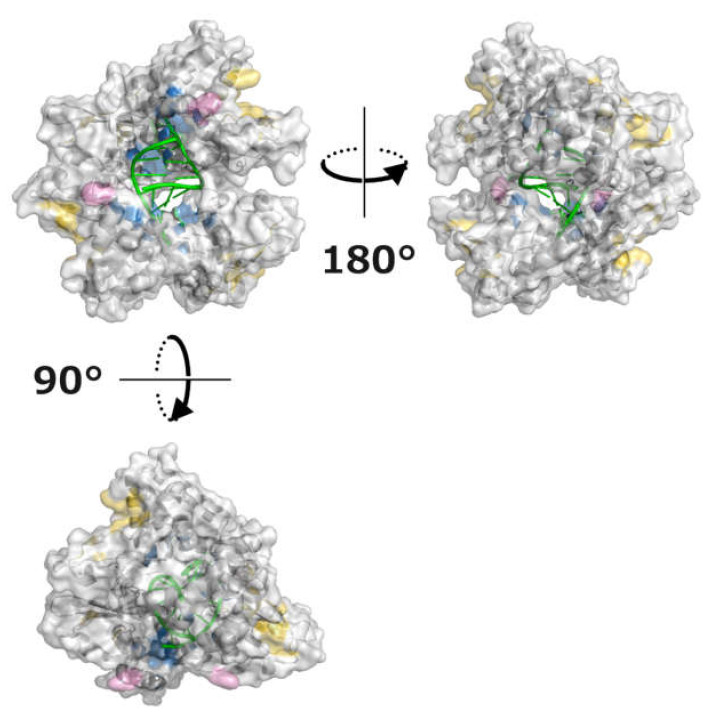
Structure of the VP35 IID-dsRNA complex. Three-dimensional structures of the VP35 IID tetramer complexed with dsRNA are shown (PDB ID: 3L26). The crystallographic asymmetric unit contains four VP35 IID molecules and one 8 base-pair dsRNA (green). Hydrophobic patch regions #1, #2, and #3 are shown in pink, blue, and yellow, respectively.

In addition to the direct interaction with dsRNA, VP35 suppresses IFN production by interacting with TBK1 and IKKε and inhibiting post-translational modifications of IRF-3/7 [6]. In this study, we were not able to clarify the detailed mechanisms by which the hydrophobic patches contribute to the suppression of IFN production. It has been reported that F239 and I278 interact with dsRNA in a van der Waals manner [16,34], suggesting that the loss of patch #2 results in a decrease in the IFN-inhibitory capacity due to decreased binding to dsRNA. On the other hand, patch #3 is located far from the interaction site of dsRNA, suggesting that the contribution of patch #3 to the suppression of IFN production may be owing to other mechanisms (Figure 5).

Previous reports have shown that MARV, LLOV, and MLAV VP35s, as well as EBOV VP35, are involved in the suppression of IRF3 activation and IFN-I production [6,35,36]. MARV VP35 also binds to dsRNA, but it recognizes a longer nucleotide length than EBOV VP35: EBOV and MARV VP35s bind to 8 bp and 18 bp of dsRNA, respectively [34]. Amino acid F239 (patch #2), which was found to affect the VP35 functions by point mutations (Figure 2 and Figure 4), has been reported to be important for hydrophobic interactions with dsRNA [16,34]. However, since F239 is highly conserved among filoviruses (Figure 1), this residue may not mainly contribute to the difference in the RNA length of dsRNA recognition between EBOV and MARV. On other hand, amino acid P293 (patch #3), which is also conserved among filoviruses and important for the VP35 functions, is located far from the dsRNA recognition site (Figure 5). The common roles of P293 in IFN antagonisms among VP35s of filoviruses need to be clarified in future studies.

Although the development of therapeutic agents for Ebola virus disease is highly desirable, drug screening using infectious filoviruses such as EBOV can only be performed in biosafety level-4 facilities. For this reason, there are many computational attempts to create viral protein-specific inhibitors against EBOV by screening more than several million compounds in silico. Inhibitors against EBOV VP35 have also been screened based on structural information [16]. Our approach will help to establish a research basis for the development of filovirus therapeutics by linking computational analyses and biological experiments that do not need to use infectious viruses.

## Figures and Tables

**Table 1:** Patch properties and effects of amino acid substitutions.

Patch	Mutation	dStability ^1^	Difference in Patch Area ^2^			Number of Patches		
			Hydrophobic	Positively Charged	Negatively Charged	Hydrophobic	Positively Charged	Negatively Charged
**#1**	F235F	0.00	0	0	0	3	8	6
	**F235A** ^3^	0.73	−100	0	0	2	8	6
	**F235G** ^3^	0.51	−100	0	0	2	8	6
	**F235S** ^3^	0.96	−100	0	0	2	8	6
	F235L ^4^	0.37	−10	0	0	3	8	6
	F235Y ^4^	0.79	−10	0	0	3	8	6
**#2**	A238A	0.00	0	0	0	3	8	6
	A238Q ^3^	1.55	−70	0	0	2	8	6
	A238P ^4^	0.56	0	0	0	3	8	6
	F239F	0.00	0	0	0	3	8	6
	**F239Q** ^3^	1.31	−70	0	0	2	8	6
	**F239N** ^3^	1.86	−70	0	0	2	8	6
	**F239H** ^3^	1.84	−70	0	0	2	8	6
	**F239K** ^3^	1.90	−70	60	0	2	9	6
	**F239A** ^3^	1.91	−20	10	0	2	8	6
	**F239Y** ^3^	0.74	−70	0	0	2	8	6
	F239V ^4^	1.83	0	0	0	3	8	6
	I278I	0.00	0	0	0	3	8	6
	**I278T** ^3^	1.66	−80	0	0	2	8	6
**#3**	P293P	0.00	0	0	0	3	8	6
	**P293N** ^3^	1.04	−110	0	0	2	8	6
	**P293D** ^3^	1.33	−110	0	0	2	8	6
	**P293Q** ^3^	0.69	−110	0	0	2	8	6
	**P293S** ^3^	1.26	−110	0	0	2	8	6
	P293I ^4^	0.02	0	0	0	3	8	6
	I295L ^4^	0.75	0	0	0	3	8	6

^1^ dStability (kcal/mol) is defined as the change in stability with no amino acid substitution as zero. ^2^ The patch area (A ^2^) of VP35 IID with the wild-type amino acid is zero, and the difference in patch area with each amino acid substitution is shown. ^3^ Mutations that were used as patch-disrupted mutants in this study (shown in boldface). ^4^ Mutations that were used as the mutants that retained the properties of the patch in this study.

## Data Availability

The data presented in this study are available on request from the corresponding author.

## Supplementary Materials

The following are available online at www.mdpi.com/article/10.3390/v13112316/s1, Table S1: Patch analysis in patch #1, Table S2: Patch analysis in patch #2, Table S3: Patch analysis in patch #3, Table S4: Summary of patch analysis, minigenome replication, NP-interaction, and IFN-β promoter-suppression data, Figure S1: Subcellular localization of VP35 mutants.
