# Supplementary material for: Functional Importance of Hydrophobic Patches on the Ebola Virus VP35 IFN-Inhibitory Domain
Source: Viruses. 2021 Nov 20;13(11):2316. doi: 10.3390/v13112316 (PMC8618116; doi:10.3390/v13112316)
Supplement: Supplementary file 1 [file viruses-13-02316-s001.zip › viruses-1431201-supplementary.pdf]

Table S1. Patch analysis in patch #1

Mutation	*dStability*[1]	Difference in patch area[2]			Number of patches		
		Hydrophobic	Positively charged	Negatively charged	Hydrophobic	Positively charged	Negatively charged
F235F	0	0	0	0	4	8	6
F235A	0.732571702	-100	0	0	3	8	6
F235R	1.006989541	-100	70	0	3	9	6
F235N	1.326652932	-90	0	40	3	8	7
F235D	1.350250176	-90	-10	80	3	8	8
F235C	0.939680112	-40	0	0	4	8	6
F235Q	0.900006362	-100	0	40	3	8	7
F235E	0.983574176	-90	0	80	3	8	7
F235G	0.505783926	-100	0	0	3	8	6
F235H	1.171110151	-100	0	80	3	8	7
F235I	0.25064059	-20	0	0	4	8	6
F235L	0.371927627	-10	0	0	4	8	6
F235K	1.080934474	-100	80	-40	3	9	6
F235M	0.431841099	-40	0	40	4	8	7
F235P	6.260009515	-90	0	40	3	8	7
F235S	0.956733005	-100	0	0	3	8	6
F235T	0.951938437	-90	0	40	3	8	7
F235W	0.781937796	-10	0	40	4	8	7
F235Y	0.78841556	-10	0	0	4	8	6
F235V	0.473775789	-30	0	40	4	8	7

[1] *dStability* (kcal/mol) is defined as the change in stability with no amino acid substitution as zero.
[2] The patch area (Å^2) of VP35 IID with the wildtype amino acid is zero, and the difference in patch area with each amino acid substitution is shown.

Table S2. Patch analysis in patch #2

Mutation	*dStability*[1]	Difference in patch area[2]			Number of patches		
		Hydrophobic	Positively charged	Negatively charged	Hydrophobic	Positively charged	Negatively charged
L232L	0	0	0	0	4	7	8
L232A	2.6275545	0	0	0	4	7	8
L232R	38.611566	-20	40	0	4	7	9
L232N	2.2629841	0	0	0	4	7	8
L232D	2.5580598	0	0	10	4	7	8
L232C	2.3945202	0	0	0	4	7	8
L232Q	2.0728772	0	0	0	4	7	8
L232E	2.0544426	0	0	0	4	7	8
L232G	3.6574852	0	0	0	4	7	8
L232H	2.8965719	0	0	-10	4	7	8
L232I	1.4270586	0	0	0	4	7	8
L232K	4.3843962	0	0	0	4	7	8
L232M	1.7285597	0	0	0	4	7	8
L232F	10.723272	20	0	0	4	7	8
L232P	29.928462	0	0	0	4	7	8
L232S	2.8143106	0	0	0	4	7	8
L232T	2.2331049	0	0	0	4	7	8
L232W	19.4083	20	0	0	4	7	8
L232Y	11.386522	-10	0	0	4	7	8
L232V	2.0320788	0	0	0	4	7	8
A238A	0	0	0	0	4	7	8
A238R	2.0994365	-70	40	0	3	7	9
A238N	0.9907217	-20	0	0	4	7	8
A238D	1.5951034	-10	0	60	4	8	8
A238C	0.8510315	0	0	0	4	7	8
A238Q	1.5488139	-70	0	0	3	7	8
A238E	2.7834175	-20	0	40	4	8	8
A238G	1.6270258	0	0	0	4	7	8
A238H	1.9547632	-20	0	60	4	8	8
A238I	5.7143486	-10	0	0	4	7	8
A238L	9.4618888	0	0	0	4	7	8
A238K	8.2175848	-20	50	0	4	7	8

A238M	1.5190067	-10	0	0	4	7	8
A238F	1.3228297	10	0	0	4	7	8
A238P	0.5634073	0	0	0	4	7	8
A238S	0.9860292	0	0	0	4	7	8
A238T	1.5008972	0	0	0	4	7	8
A238W	2.0445495	40	0	0	4	7	8
A238Y	1.1203081	-10	0	0	4	7	8
A238V	2.5027563	-10	0	0	4	7	8
F239F	0	0	0	0	4	7	8
F239A	1.9057251	-20	10	0	4	7	8
F239R	1.6876262	-80	60	0	3	7	9
F239N	1.8570417	-70	0	0	3	7	8
F239D	2.1112167	-80	0	0	3	7	8
F239C	1.8044742	-30	0	0	4	7	8
F239Q	1.3112751	-70	0	0	3	7	8
F239E	1.760937	-70	0	0	3	7	8
F239G	2.81543	-30	10	0	4	7	8
F239H	1.838075	-70	0	0	3	7	8
F239I	2.1238505	-10	0	0	4	7	8
F239L	0.7491947	-10	0	0	4	7	8
F239K	1.8976756	-70	60	0	3	7	9
F239M	1.3301483	-10	0	0	4	7	8
F239P	6.9946532	-10	0	0	4	7	8
F239S	2.1576557	-80	0	0	3	7	8
F239T	1.7906735	-30	0	0	4	7	8
F239W	1.4098491	10	0	0	4	7	8
F239Y	0.7401587	-70	0	0	3	7	8
F239V	1.8329102	0	0	0	4	7	8
Q274Q	0	0	0	0	3	7	8
Q274A	0.9790117	60	0	0	4	7	8
Q274R	0.6115132	50	60	0	4	7	9
Q274N	1.0520914	50	0	0	4	7	8
Q274D	1.3061332	50	0	0	4	7	8
Q274C	0.8424939	100	0	0	4	7	8
Q274E	1.0742681	60	0	0	4	7	8
Q274G	1.565205	70	10	0	4	7	8

Q274H	1.0874786	50	0	0	4	7	8
Q274I	0.824607	110	0	0	4	7	8
Q274L	0.1510161	120	0	0	4	7	8
Q274K	0.9377139	40	70	0	4	7	9
Q274M	0.3732078	110	0	0	4	7	8
Q274F	0.8585927	120	0	0	4	7	8
Q274P	56.740468	100	0	0	4	7	8
Q274S	1.2294898	50	0	0	4	7	8
Q274T	0.8657571	90	0	0	4	7	8
Q274W	0.9901868	140	0	0	4	7	8
Q274Y	1.2227983	120	0	0	4	7	8
Q274V	0.5805623	110	0	0	4	7	8
I278I	0	0	0	0	4	7	8
I278A	1.9591054	-10	40	0	4	7	8
I278R	1.3934811	-90	70	-10	3	7	9
I278N	1.9687262	-80	40	0	3	7	9
I278D	2.2570228	-80	0	60	3	8	8
I278C	1.9057499	-10	40	0	4	7	9
I278Q	1.6894351	-80	40	0	3	7	9
I278E	1.6783633	-80	0	40	3	8	8
I278G	2.752968	-30	10	0	4	7	8
I278H	1.9107519	-80	0	40	3	8	8
I278L	0.8558835	-10	0	0	4	7	8
I278K	1.5554021	-30	50	0	4	7	9
I278M	0.9461162	-30	0	0	4	7	8
I278F	1.1611346	-20	0	0	4	7	8
I278P	153.5848	-10	0	0	4	7	8
I278S	2.1471819	-80	40	0	3	7	9
I278T	1.6575709	-80	0	0	3	7	8
I278W	1.5097841	-10	0	-40	4	6	8
I278Y	1.2976086	-30	0	0	4	7	8
I278V	1.0096868	-10	0	0	4	7	8

[1] *dStability* (kcal/mol) is defined as the change in stability with no amino acid substitution as zero.
[2] The patch area (Å^2) of VP35 IID with the wildtype amino acid is zero, and the difference in patch area with each amino acid substitution is shown.

Table S3. Patch analysis in patch #3

Mutation	*dStability*[1]	Difference in patch area[2]			Number of patches		
		Hydrophobic	Positively charged	Negatively charged	Hydrophobic	Positively charged	Negatively charged
V245V	0	0	0	0	110	4	7
V245A	1.94009808	-10	0	0	4	7	8
V245R	1.73968582	-110	30	0	3	7	8
V245N	1.96042938	-10	0	0	4	7	8
V245D	2.16483607	-10	0	0	4	7	8
V245C	1.804856894	0	0	0	4	7	8
V245Q	2.52690014	0	0	0	4	7	8
V245E	4.685006891	-60	0	40	4	8	8
V245G	2.939590088	-10	0	0	4	7	8
V245H	10.29826373	-10	0	0	4	7	8
V245I	1.420383087	0	0	0	4	7	8
V245L	34.03914758	0	0	0	4	7	8
V245K	37.98844083	-110	0	0	3	7	8
V245M	2.76672884	0	0	0	4	7	8
V245F	825.8513853	-10	0	0	4	7	8
V245P	588.9697724	0	0	0	4	7	8
V245S	2.085385378	-10	0	0	4	7	8
V245T	2.209940464	-10	0	0	4	7	8
V245W	72.62961212	10	0	0	4	7	8
V245Y	95887186.48	-10	0	0	4	7	8
K248K	0	0	0	0	4	7	8
K248A	0.095168878	-10	-40	40	4	8	8
K248R	-0.519800474	-100	-30	40	3	8	8
K248N	0.28260355	-10	-50	40	4	8	8
K248D	0.6330468	-50	-40	70	4	8	8
K248C	0.085650141	-10	-40	40	4	8	8
K248Q	0.29007365	-10	-40	0	4	7	8
K248E	0.532215671	-10	-50	70	4	8	8
K248G	0.552775281	-10	-40	40	4	8	8
K248H	0.291377256	-10	-50	90	4	9	8
K248I	-0.68891395	40	-30	0	4	7	8
K248L	-0.640784223	30	-40	0	4	7	8

K248M	-0.248851966	10	-40	40	4	8	8
K248F	0.100037599	50	-40	0	4	7	8
K248P	981.5469749	-10	-40	40	4	8	8
K248S	0.242675293	-10	-40	40	4	8	8
K248T	0.006153339	-10	-40	0	4	7	8
K248W	-0.990039757	80	-20	40	5	8	8
K248Y	-0.814420669	20	-30	40	4	8	8
K248V	-0.391183967	20	-40	0	4	7	8
L249L	0	0	0	0	4	7	8
L249A	2.055125935	-20	0	0	4	7	8
L249R	1.255585451	-30	0	-10	4	7	8
L249N	1.910848759	-30	0	0	4	7	8
L249D	1.984240956	-50	0	40	4	8	8
L249C	1.941255393	0	0	0	4	7	8
L249Q	1.744715848	-40	0	-10	4	7	8
L249E	1.946672273	-40	0	30	4	7	8
L249G	2.884809407	-20	0	0	4	7	8
L249H	1.687257971	-30	0	30	4	7	8
L249I	1.278723454	0	0	0	4	7	8
L249K	1.786890975	-20	0	-10	4	7	8
L249M	1.29713662	-20	0	0	4	7	8
L249F	0.750303334	10	0	0	4	7	8
L249P	432.4197974	0	0	0	4	7	8
L249S	2.255533647	-50	0	0	4	7	8
L249T	2.204444709	-10	0	0	4	7	8
L249W	0.885336346	20	0	0	4	7	8
L249Y	0.834071571	0	0	0	4	7	8
L249V	1.628334062	0	0	0	4	7	8
A290A	0	0	0	0	4	7	8
A290R	0.504601777	-10	70	-30	4	7	9
A290N	0.723215064	-20	0	-10	4	7	8
A290D	1.120206587	-20	0	50	4	7	8
A290C	0.796192933	0	0	-10	4	7	8
A290Q	0.86041541	-20	0	20	4	7	8
A290E	0.987338204	-20	0	50	4	7	8
A290G	1.482704634	0	0	0	4	7	8

A290H	1.361392088	-20	0	40	4	7	8
A290I	5.390960481	20	0	-10	4	7	8
A290L	-0.123682767	20	10	-10	4	7	8
A290K	1.037420222	-30	70	-10	4	7	9
A290M	0.526912091	20	0	0	4	7	8
A290F	0.542257567	40	0	-10	4	7	8
A290P	1125.794701	0	0	-10	4	7	8
A290S	1.023275747	-20	0	-10	4	7	8
A290T	0.941858651	-10	0	0	4	7	8
A290W	1.004867449	60	0	-10	4	7	8
A290Y	0.616011644	20	10	-10	4	7	8
A290V	3.577328926	0	0	-10	4	7	8
P293P	0	0	0	0	4	7	8
P293A	1.067504566	-10	0	0	4	7	8
P293R	0.133816822	-110	40	0	3	7	8
P293N	1.035976962	-110	0	0	3	7	8
P293D	1.328013011	-110	0	0	3	7	8
P293C	1.023459389	-10	0	0	4	7	8
P293Q	0.68896101	-110	0	0	3	7	8
P293E	0.707244618	-110	-10	0	3	7	8
P293G	1.856066142	-30	0	0	4	7	8
P293H	1.538806997	-20	-10	0	4	7	8
P293I	0.019388362	0	0	0	4	7	8
P293L	-0.054958234	10	0	0	4	7	8
P293K	1.052286242	-50	50	0	4	7	8
P293M	0.207329841	0	0	0	4	7	8
P293F	0.85928369	60	0	0	4	7	8
P293S	1.262504563	-110	0	0	3	7	8
P293T	0.758316384	-60	0	0	4	7	8
P293W	0.894564793	20	0	0	4	7	8
P293Y	0.963431749	30	0	0	4	7	8
P293V	0.022220093	0	0	0	4	7	8
I295I	0	0	0	0	4	7	8
I295A	2.023302934	-10	0	-40	4	6	8
I295R	0.859431366	-110	10	0	3	7	8
I295N	1.99553434	-50	0	0	4	7	8

I295D	2.253890578	-60	0	60	4	8	8
I295C	2.039158126	-40	0	-40	4	6	8
I295Q	1.479317877	-50	0	0	4	7	8
I295E	1.916342451	-50	0	40	4	8	8
I295G	2.763974997	-20	0	-40	4	6	8
I295H	1.957786738	-50	0	50	4	8	8
I295L	0.751394736	0	0	0	4	7	8
I295K	1.623988688	-50	40	0	4	7	9
I295M	1.176933426	-10	0	0	4	7	8
I295F	0.704692173	0	0	0	4	7	8
I295P	82.9752419	-10	0	0	4	7	8
I295S	2.240752554	-40	0	0	4	7	8
I295T	1.789046233	-50	0	0	4	7	8
I295W	0.879898913	0	0	0	4	7	8
I295Y	0.974483428	-10	0	0	4	7	8
I295V	1.08541018	-10	0	0	4	7	8
I297I	0	0	0	0	4	7	8
I297A	2.217895682	-10	0	-40	4	6	8
I297R	2.275249985	-30	0	0	4	7	8
I297N	2.026114101	-10	0	0	4	7	8
I297D	2.536684628	-10	0	-40	4	6	8
I297C	2.030322076	-10	0	-40	4	6	8
I297Q	2.05753488	-20	0	0	4	7	8
I297E	2.390634188	-20	0	80	4	8	8
I297G	3.133241422	-10	10	-40	4	6	8
I297H	2.838109053	-20	0	70	4	8	8
I297L	1.592453204	0	0	0	4	7	8
I297K	2.991622117	-30	0	0	4	7	8
I297M	1.846678482	-10	0	0	4	7	8
I297F	2.136438321	10	0	0	4	7	8
I297P	23.19278575	-10	0	-40	4	6	8
I297S	2.408419279	-10	0	-40	4	6	8
I297T	1.925662195	-10	0	-40	4	6	8
I297W	3.220605793	40	0	-40	4	6	8
I297Y	1.83253497	0	0	0	4	7	8
I297V	1.132516364	-10	0	0	4	7	8

F328F	0	0	0	0	4	7	8
F328A	2.983770293	30	0	0	4	7	8
F328R	1.92821213	-20	0	0	4	7	8
F328N	2.841497587	-10	0	0	4	7	8
F328D	2.925697332	-10	0	0	4	7	8
F328C	2.868945471	20	0	0	4	7	8
F328Q	2.543589049	0	0	0	4	7	8
F328E	2.859739658	-20	0	0	4	7	8
F328G	3.985298561	40	0	-40	4	6	8
F328H	2.421494646	-10	0	0	4	7	8
F328I	22.58227036	10	0	0	4	7	8
F328L	1.980802214	0	0	0	4	7	8
F328K	2.331466483	-20	0	0	4	7	8
F328M	1.87006816	-10	0	0	4	7	8
F328P	119.9410066	30	0	0	4	7	8
F328S	3.206147808	0	0	0	4	7	8
F328T	2.506706817	20	0	0	4	7	8
F328W	1.162626502	0	0	0	4	7	8
F328Y	0.941289061	0	0	0	4	7	8
F328V	2.672337747	20	0	0	4	7	8

[1] *dStability* (kcal/mol) is defined as the change in stability with no amino acid substitution as zero.
[2] The patch area (Å^2) of VP35 IID with the wildtype amino acid is zero, and the difference in patch area with each amino acid substitution is shown.

Table S4: Summary of patch analysis, minigenome replication, NP-interaction, and IFN-β promoter-suppression data

Hydrophobic patch	Effect on patch	VP35	Minigenome replication	Interaction with NP	Suppression of IFN-β promoter
#1		Wildtype	++	+	+++
	Disrupted	R225E	-	ND[1]	+++
		F235A	+++	ND	+++
		F235G	++	+	+++
		F235S	++	ND	+++
	**Retained**[2]	**F235L**	++	ND	+++
		**F235Y**	++	ND	+++
#2	Disrupted	A238Q	++	+	±
	**Retained**	**A238P**	+++	ND	+++
	Disrupted	F239Q	++	ND	-
		F239N	+++	ND	-
		F239H	++	ND	-
		F239K	-	-	-
		F239Y	++	+	+++
		F239A	++	ND	-
	**Retained**	**F239V**	++	ND	-
	Disrupted	I278T	++	ND	+++
#3	Disrupted	P293N	-	-	±
		P293D	-	-	±
		P293Q	-	-	±
		P293S	±	-	-
	**Retained**	**P293I**	-	ND	-
		**I295L**	±	ND	-

[1]ND: Not done

[2]Patch-retained mutants are shown in boldface.

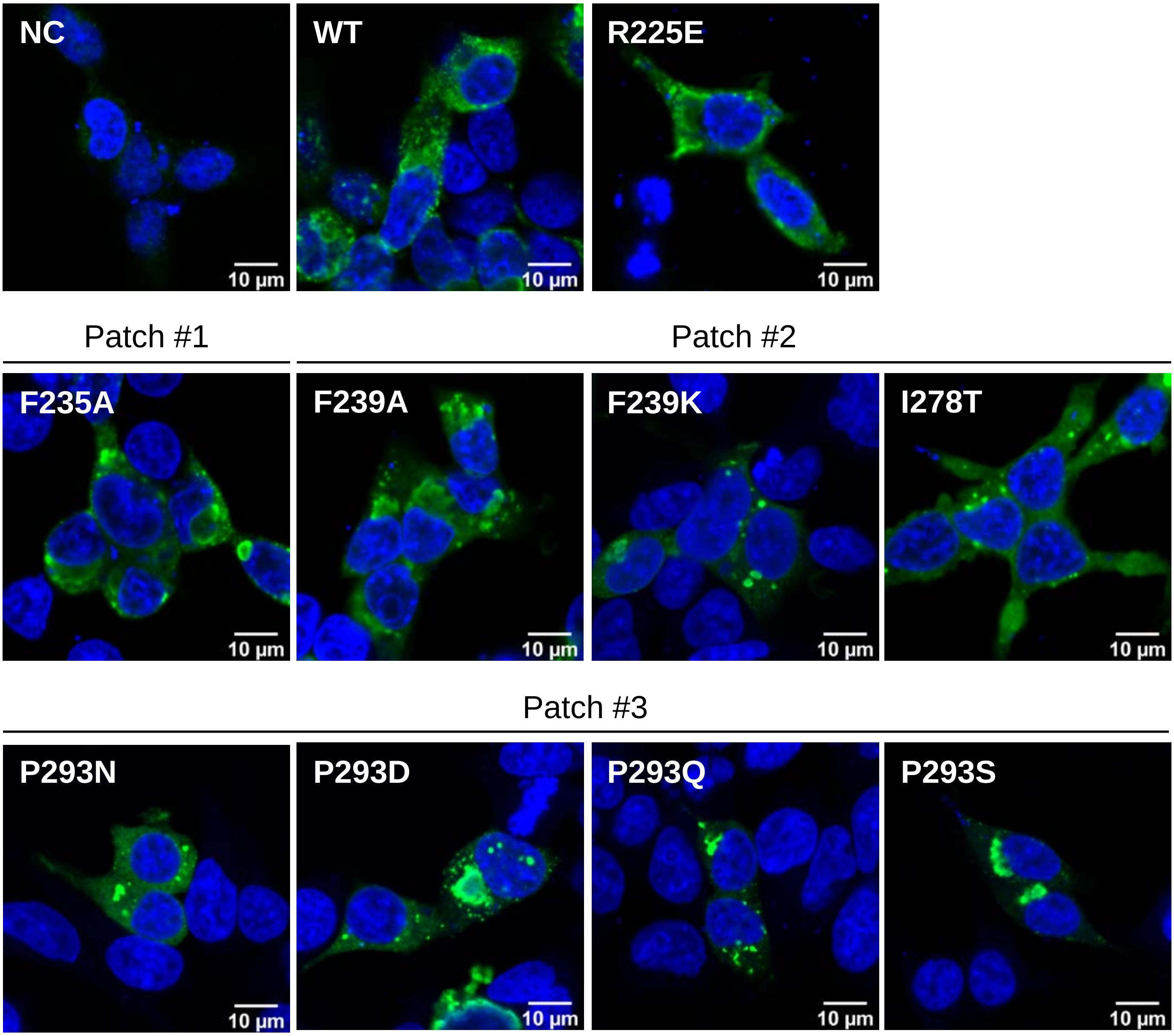

Figure S1: Subcellular localization of VP35 mutants

Subcellular localization of VP35 was confirmed by immunofluorescence analysis. HEK293T cells were transfected with an empty vector (NC), WT, or mutant VP35-expressing plasmids. HA-tagged WT or VP35 mutants were detected with a monoclonal anti-HA antibody (Abcam) and Alexa488-labeled anti-mouse IgG (H+L)(Life Technologies). DAPI (blue) was used for nuclear staining.
